# Toward a Complete North American *Borrelia miyamotoi* Genome

**DOI:** 10.1128/genomeA.01557-16

**Published:** 2017-02-02

**Authors:** Luke C. Kingry, Adam Replogle, Dhwani Batra, Lori A. Rowe, Christopher Sexton, Marc Dolan, Neeta Connally, Jeannine M. Petersen, Martin E. Schriefer

**Affiliations:** a Division of Vector-Borne Diseases, Bacterial Diseases Branch, Centers for Disease Control and Prevention, Fort Collins, Colorado, USA; b Division of Scientific Resources, Biotechnology Core Facility Branch, Centers for Disease Prevention and Control, Atlanta, Georgia, USA; c Department of Biological and Environmental Sciences, Western Connecticut State University, Danbury, Connecticut, USA

## Abstract

*Borrelia miyamotoi*, of the relapsing-fever spirochete group, is an emerging tick-borne pathogen causing human illness in the northern hemisphere. Here, we present the chromosome, eight extrachromosomal linear plasmids, and a draft sequence for five circular and one linear plasmid of a *Borrelia miyamotoi* strain isolated from an *Ixodes* sp. tick from Connecticut, USA.

## GENOME ANNOUNCEMENT

*Borrelia miyamotoi*, a member of the relapsing-fever *Borrelia* group, is widely distributed in *Ixodes* sp. ticks throughout the northern hemisphere (1). The public health impact due to *B. miyamotoi* infection has been highlighted by recent reports of human disease in Europe, Russian Federation, Japan, and the United States (2–5). Unlike most relapsing fever spirochetes that are transmitted by soft-bodied argasid ticks, *B. miyamotoi* is transmitted by hard-bodied ixodid ticks, which also transmit Lyme disease–causing *Borrelia* spp. (6). The *B. miyamotoi* genome consists of a large linear chromosome (~1 Mb) and an unknown number of extrachromosomal linear and circular plasmids (7). Sequence data for *B. miyamotoi* plasmids currently exist in unfinished raw contig form. The assembly of plasmids is complicated by the presence of long repetitive and paralogous sequences (8, 9).

Pure culture of *B. miyamotoi* CT13-2396 was achieved from a single *Ixodes scapularis* nymph reared from the egg clutch of an infected adult female collected in 2013 from Connecticut, USA. Genomic DNA was extracted from cultured *B. miyamotoi* using the QIAamp DNA kit (Qiagen, Valencia, CA, USA). Sequencing was performed at the CDC Genome Sequencing Lab (Atlanta, GA, USA) using the Pacific Biosciences RSII instrument, with one single-molecule real-time cell, and assembled with the Hierarchal Genome Assembly Process (HGAP3) (Pacific Biosciences, Menlo Park, CA, USA; C3 chemistry, movie time = 145 min) (10). The average read length was 5,516 bp, with a mean read score of 0.86 and average coverage of 264×. Sequence trimming, stitching, and resequencing analysis were performed as previously described to validate topology (8). Annotation was performed using the Prokaryotic Genome Annotation Pipeline (11).

The complete 907,278-bp *B. miyamotoi* CT13-2396 linear chromosome (average G+C 28.7%) is predicted to encode 841 open reading frames (ORFs), three rRNAs, 31 tRNAs, and 19 pseudogenes. The reported genome includes eight complete linear plasmids (lp72, lp41, lp30, lp23, lp20-1, lp20-2, lp19, and lp6) ranging in size from 6 kb to 72 kb and totaling 234,563 bp. Incomplete plasmids include up to five putative circular plasmids (cp1 to cp5) and one linear plasmid (lp26) totaling 135,554 bp, ranging in size from 17 kb to 29 kb. The average G+C content of the total plasmid DNA is 28.5%. Notably, 23 ORFs were identified in the CT13-2396 plasmids, which encode for variable large proteins (Vlp’s). Comparison of these Vlp protein sequences revealed a conserved 26–amino acid C-terminal peptide homologous to the C6 peptide in the VlsE protein from *B. burgdorferi*, the causative agent of Lyme disease. Alignment of the 23 Vlp peptide sequences to the *B. burgdorferi* B31 C6 peptide demonstrated seven to 16 identical amino acids and seven to 11 conservative amino acid substitutions. This finding is consistent with a previous report describing C6 seropositivity in a *B. miyamotoi*–infected patient (12).

The draft sequence for *B. miyamotoi* CT13-2396 described here provides a valuable addition toward the completion of a full *B. miyamotoi* genome and a resource for studying the genomics and pathogenesis of this emerging human pathogen.

### Accession number(s).

The *B. miyamotoi* CT13-2396 genome sequence (chromosome and plasmids) is available from GenBank under the accession numbers CP017126 to CP017140.
